# The Current State of MicroRNAs as Restenosis Biomarkers

**DOI:** 10.3389/fgene.2019.01247

**Published:** 2020-01-10

**Authors:** Nelson Varela, Fernando Lanas, Luis A. Salazar, Tomás Zambrano

**Affiliations:** ^1^ Laboratory of Chemical Carcinogenesis and Pharmacogenetics, Department of Basic-Clinical Oncology, Faculty of Medicine, Universidad de Chile, Santiago, Chile; ^2^ Department of Internal Medicine, Faculty of Medicine, Universidad de La Frontera, Temuco, Chile; ^3^ Center of Molecular Biology and Pharmacogenetics, Scientific and Technological Bioresource Nucleus, Universidad de La Frontera, Temuco, Chile; ^4^ Department of Medical Technology, Faculty of Medicine, Universidad de Chile, Santiago, Chile

**Keywords:** epigenetics, microRNAs, in-stent restenosis, biomarkers, personalized and precision medicine

## Abstract

In-stent restenosis corresponds to the diameter reduction of coronary vessels following percutaneous coronary intervention (PCI), an invasive procedure in which a stent is deployed into the coronary arteries, producing profuse neointimal hyperplasia. The reasons for this process to occur still lack a clear answer, which is partly why it remains as a clinically significant problem. As a consequence, there is a vigorous need to identify useful non-invasive biomarkers to differentiate and follow-up subjects at risk of developing restenosis, and due to their extraordinary stability in several bodily fluids, microRNA research has received extensive attention to accomplish this task. This review depicts the current understanding, diagnostic potential and clinical challenges of microRNA molecules as possible blood-based restenosis biomarkers.

## Introduction

Cardiovascular disease (CVD) refers to a group of pathologies initiated by an underlying process known as atherosclerosis and ultimately affecting the heart and blood vessels. Atherosclerosis plaques build-up inside the coronary arteries, consequently limiting the blood flow and resulting in coronary artery disease (CAD). Atherosclerosis is an inflammatory disease (Ross, 1999) able to produce two morphologically opposite lesions within the coronary arteries, stenotic and non-stenotic. The last may be asymptomatic for years and clinical management is generally supported on lifestyle modifications and, eventually, pharmacological interventions in high-risk individuals. In contrast, stenotic lesions have clinical manifestations like angina pectoris, and common medical management includes revascularization procedures such as coronary artery bypass grafting (CABG) and percutaneous transluminal coronary angioplasty (PTCA), a widely performed techniques since the late 1970s to correct serious coronary atherosclerotic lesions (Gruntzig et al., 1979), restoring myocardial blood flow and reducing angina symptoms. Despite its massive use, however, elevated restenosis rates affected almost half of the patients treated (Fischman et al., 1994) and established one of the main problems of current cardiology. Restenosis is arbitrarily defined as a narrowing of vessel diameter greater than 50% to that of the reference vessel (Marx et al., 2011), and results from excessive proliferation and migration of vascular smooth muscle cells (VSMC) to the intima, eventually leading to re-narrowing of the arterial lumen (Chaabane et al., 2013). Since this problem was identified, interventional cardiology has moved from PTCA to percutaneous coronary intervention (PCI), a technique involving the placement of a stent. This procedure is the most widely performed treatment for symptomatic coronary disease patients (Serruys et al., 1994). The use of bare metal stents (BMS) has made it possible to eliminate factors that favor restenosis, such as elastic recoil and negative remodeling (Lowe et al., 2002), reducing the prevalence of restenosis from 50% to 20-30% (Kastrati et al., 1997). As the main cause of restenosis was attributed to the excessive proliferation of VSMC, the development of new technologies determined the arrival of drug-eluting stents (DES), reducing the restenosis rate below 10% (Morice et al., 2002). Despite the implementation of new stenting technologies, along with novel pharmacological or mechanical approaches to reduce restenosis incidence, this problem is still considered an important drawback, especially in high-risk patients, limiting the overall success of DES.

## Pathophysiology of Restenosis in Stented Arteries

Stent placement produces a mechanical vascular lesion that can be briefly divided into 3 phases:

- Early phase: the stent produces an injury to the endothelium, damaging or totally destroying the endothelial cells (EC) that line the intimal arterial tunic, resulting in consecutive endothelial stripping, re-endothelization and subsequent generation of neo-endothelium (Grewe et al., 2000). The above is followed by an inflammatory response, including platelet activation and recruitment of circulating leukocytes, releasing cytokines and growth factors (Mitra and Agrawal, 2006).
- Intermediate phase: characterized by the migration and proliferation of VSMC.
- Late phase or tissue remodeling: VSMCs change from a contractile and quiescent non-proliferative G0 phase phenotype towards a highly active synthetic phenotype, with extracellular matrix (ECM) deposition in the arterial intima. Various growth factors, such as fibroblast growth factor (FGF-2), epidermal growth factor (EGF), platelet-derived growth factor (PDGF), and insulin-like growth factor (IGF) initiate VSMCs proliferation through the tyrosine kinase receptor, activating the Mitogen-activated protein kinases (MAPK) pathway. While the ECM allows the inflammatory infiltrate to adhere, the VSMC secrete hyaluronic acid and proteoglycans that interact and stabilize the fibrin-enriched ECM (Grewe et al., 2000; Mitra and Agrawal, 2006). These vascular responses, characterized by neointima proliferation and vascular remodeling, are responsible for the elevated frequency of post-PTCA restenosis. Anatomopathological studies in post-PCI restenosis demonstrated the same proliferative response of the neointima (Farb et al., 2002; Farb et al., 2004). In addition to VSMC proliferation and ECM synthesis, there is also neointima colonization by extravascular cells, e.g., endothelial progenitors or dendritic cells, together with compensatory mechanisms of apoptosis (Tuleta et al., 2008; Tuleta et al., 2010).

## Types of Stent: Bare Metal Stent and Drug-Eluting Stent

Since the introduction of BMS in 1987 (Sigwart et al., 1987), important PTCA limitations such as restenosis and sudden narrowing of diseased arteries after angioplasty were reduced. Serruys et al. demonstrated that stent implantation reduces the need for a second coronary angioplasty compared with standard balloon angioplasty (RR 0.58, CI 0.40–0.85) mainly due to a low restenosis rate, going from 32% to 22% (Serruys et al., 1994). However, the benefit was accompanied by an increased risk of cardiovascular complications and longer hospitalization time. Stent-induced injury causes greater damage than damaged produced by standard balloon angioplasty, delineating processes of thrombosis, inflammation and proliferation (Edelman and Rogers, 1998) followed by the deposition of platelet-rich thrombi, which occur from the first days (Farb et al., 1999) until 1 month post-PCI (Komatsu et al., 1998), with additional accumulation of acute inflammatory cells such as neutrophils during the first 30 days, together with chronic inflammatory cells e.g., lymphocytes and macrophages (Farb et al., 1999). There is a correlation between the type of inflammatory reaction and the degree of injury (Rogers and Edelman, 1995), indicating that the surface of the material together with the geometric configuration of the stent contributes to neointimal hyperplasia and thrombosis. Other factors that favor restenosis development are tunica media damage, and the penetration of the stent edges into the lipid core of the atherosclerotic plaque. Both factors increase the inflammatory process within the artery and, therefore, increase intima proliferation (Farb et al., 2002). Since the introduction of combination therapy with P2Y platelet receptor antagonists (ticlopidine, clopidogrel) and acetylsalicylic acid, the incidence of post-stent thrombosis has been significantly reduced (Bertrand et al., 2000), and the majority of thrombotic events occurred within the first 10 days post-PCI. Additionally, post-stent thrombosis with BMS after the first month is considered rare (Farb et al., 2003). DES development is based on a coating containing an antiproliferative drug, so both post-PCI proliferation of the tunica intima and subsequent restenosis can be reduced (Garg and Serruys, 2010). In this way, first-generation DES devices were developed, which are coated with a drug-containing polymer designed to interrupt cell replication and reduce neointimal hyperplasia, markedly decreasing the occurrence of post-PCI restenosis (Herdeg et al., 2000; Curfman, 2002). However, after DES implantation, intervention centers have identified an increase in thrombosis associated with the stent placement process for up to 3 years after stent implantation, an additional complication rarely caused by the use of BMS (Luscher et al., 2007). Several reports show the occurrence of acute (<24 hours), sub-acute (<30 days), late (>30 days), and very late (>12 months) thrombosis after DES placement (McFadden et al., 2004; Pfisterer et al., 2006; Brodie et al., 2012). A large observational study revealed that from a total of 2229 consecutive patients receiving a total of 4495 DES, 29 had stent- associated thrombosis, occurring more than 30 days after stent placement, also revealing a 45% mortality rate (Iakovou et al., 2005).

## MicroRNAs

In 1993, a key report involving the study of the *Caenorhabditis elegans* roundworm and showing downregulation of the LIN-14 protein by a small transcript namely lin-4 through antisense interaction between RNAs due to sequence complementarity between lin-4 and the 3'-untranslated region (3'-UTR) of the lin-14 mRNA (Lee et al., 1993) suggested a novel gene silencing mechanism affecting protein levels. Afterward, a second 21 nucleotides (nt) small RNA identified as let-7 was also implicated in the regulation of heterochronic genes related to *C. elegans* development (Reinhart et al., 2000). Moreover, the small let-7 RNA was shown to be highly conserved, indicating that its sequence is critical for functional purposes (Pasquinelli et al., 2000). These small RNAs were the 2 first of a family currently known as microRNAs (miRNAs) and further characterized as endogenous non-coding RNAs (ncRNAs) evolutionarily conserved between species with a size comprised between 20 and 23 nt, existing in both plants and animals. Their main function is to control gene expression by cleaving messenger RNA (mRNA) or through translational repression, preventing mRNA translation to its corresponding protein (Bartel, 2004). This control mechanism fine-tunes gene expression through the complementary matching of a segment comprised between nucleotides 2 to 7 of the miRNA i.e., seed region, with both the 3′- and 5′-UTR regions of target mRNAs (Lytle et al., 2007). It is estimated that miRNAs control more than 30% of the human genome (Lewis et al., 2005), through an interaction that can be reversible (Wu and Belasco, 2008). The canonical pathway of miRNA biogenesis begins with transcription from miRNA genes by RNA polymerase II, producing primary miRNAs (pri-miRNA) that undergo subsequent processing by the Drosha-DGCR8 (DiGeorge syndrome critical region 8) microprocessor complex, producing a miRNA precursor (pre-miRNA) of approximately 70 nt transported to the cytoplasm *via* exportin 5 (XPO5). Once in the cytoplasm, pre-miRNAs are further processed by RNase III (Dicer) into a double-stranded 21-23 nt miRNA. One strand of the miRNA is charged into the RNA-induced silencing complex (RISC) in conjunction with members of the Argonaute protein family (AGO2), a nuclear protein essential for miRNA maturation and functionality (Bushati and Cohen, 2007; Winter et al., 2009).

**Figure 1:**
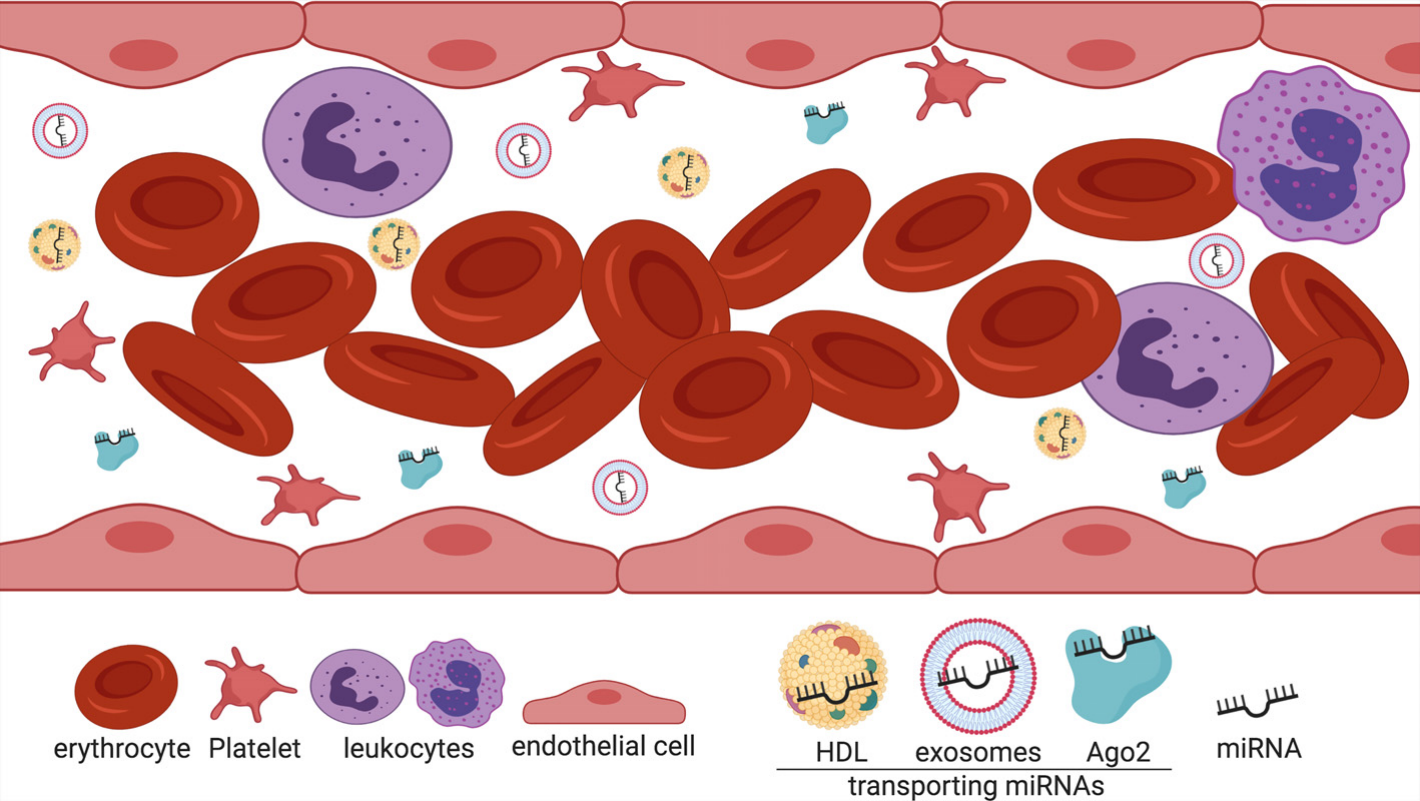
Extracellular miRNAs are associated with different transport molecules in the bloodstream. Cell-free miRNAs are transported in the blood mainly associated with Ago2 (green) and HDL particles (yellow). To a lesser extent, miRNAs are transported *via* exosomes (light blue). Created with BioRender.

Different studies show that miRNAs orchestrate a wide network of cellular activities and are deeply involved in almost every biological pathway, regulating processes such as cell division and apoptosis (Ng et al., 2012), metabolism (Wilfred et al., 2007), intracellular signaling (Zhang et al., 2012), immune response (Taganov et al., 2006) and cell movement (Png et al., 2011). Similarly, miRNAs have been associated with restenosis-related processes, such as VSMC proliferation, migration and neointima formation (Chen et al., 2012; Yamakuchi, 2012; Gareri et al., 2016), revealing the great potential for diagnostic, prognostic, therapeutics or additional clinical manipulation. In fact, by examining the hypothesis that miRNAs produced by the placenta can be released into circulation, a set of placental miRNAs was successfully identified in maternal plasma (Chim et al., 2008), shedding light into another possible role as blood-based biomarkers, a crucial finding confirmed during the same year by a meticulous characterization of a large number of exceptionally stable miRNAs in both serum and plasma (Chen et al., 2008b). Since then, numerous reports have shown that miRNAs can be detectable in multiple fluids including urine, saliva, and cerebrospinal fluid, and even though the extracellular environment is rich in ribonucleases (RNases), miRNAs can be especially stable in serum and plasma as well, representing an enormous potential as non-invasive biomarkers for several pathologies (Gilad et al., 2008; Mitchell et al., 2008; Gupta et al., 2010). The mechanisms by which miRNAs remain particularly unaffected in circulation are due to their association with different carrier particles that confer protection against the potent blood RNases (**Figure 1**). It was first proposed that miRNAs circulate in the bloodstream by a cellular discharge mechanism through membrane-bound vesicles such as exosomes (Valadi et al., 2007; Kosaka et al., 2010), which are 50 to 100 nm vesicles released by exocytosis (Fevrier and Raposo, 2004). However, reports indicated that the abundant majority of miRNAs are exosome free and associated with Ago2 (Arroyo et al., 2011; Turchinovich et al., 2011). Importantly, as the RISC constitutes the effector component of the gene-silencing mechanism portrayed by miRNAs, it has been suggested that the miRNA-Ago2 complex is functional in circulation. Moreover, in 2011, Vickers et al. showed that miRNAs are associated with HDL in plasma not only for transport but these complexes maintain also the functional gene repression role of miRNAs directed to their cell target through delivery by a scavenger receptor BI (SR-BI)-dependent mechanism (Vickers et al., 2011). Considering that miRNAs are associated with dissimilar transport molecules, further classification of extracellular miRNAs according to their transporting molecules has been provided elsewhere (Russo et al., 2012).

## Biomarker Discovery

In general, biomarkers are classified as: (1) Diagnostic biomarkers for a specific pathology, disease or syndrome; (2) predictive biomarkers for the response to a given medication or treatment; (3) biomarkers of predictions about the probable course of a disease; and (4) biomarkers of predisposition or susceptibility to a disease (Simon, 2011). According to the World Health Organization, a biomarker is "any substance, structure, process or products that can be measured in the body and influence or predict the outcome or incidence of the disease". Different study models have used cell lines, animals, patient cohorts, biopsies, biobank samples, or prospective studies as the starting point for biomarker development (Vargas and Harris, 2016). On the other hand, "omics" technologies are a particularly suitable tool for biomarkers discovery as they take advantage of the potential of the transcriptome, proteome and metabolome readings, facilitating detailed molecular characterization in a particular biological sample. Examples of these technologies are microarrays and next-generation sequencing (NGS) used for genomic and transcriptomic studies. In this sense, the usual strategy has been to describe large amounts of data from a specific molecule (e.g., miRNAs), in samples such as cell lines, animals and most importantly patients with a specific condition (McShane and Polley, 2013; Ghai and Wang, 2016) in order to generate hypotheses based on the large data available following bioinformatic analysis (Simon, 2010). Thus, new proposed biomarkers are capable, for example, of facilitating diagnosis of a certain disease or predicting the response of therapeutic interventions, such as post-stenting restenosis. Currently, a number of reasons have proposed circulating miRNAs as one of the most attractive candidates molecules to be explored as diagnosis, prognosis, and treatment biomarkers for various pathologies, mainly their extraordinary stability in blood circulation, the relative ease of extraction from the most common non-invasive matrices, and their susceptibility to sensitive detection through quantitative polymerase chain reaction (qPCR) (Gilad et al., 2008; Moldovan et al., 2014; Ghai and Wang, 2016) and rapid multiplexing platforms (Jiang et al., 2014).

## Circulating Mirnas as Restenosis Biomarkers

Few investigations have examined the utility of cell-free miRNAs as potential in-stent restenosis (ISR) biomarkers. One of the pioneer reports was a case-control study revealing a series of 4 miRNAs ‑miRNA-21, miRNA-100, miRNA-143 and miRNA-145 ‑ as candidate ISR markers, with the two latter showing the highest sensitivity and specificity according to receiver operating characteristic (ROC) curves (He et al., 2014) (**Table 1**). Consistent with their newfound role, these 4 miRNAs have been previously related to the pathogenesis of vascular diseases such as neointimal lesion formation (Ji et al., 2007), and VSMC proliferation, migration, and differentiation (Davis et al., 2008; Cordes et al., 2009; Grundmann et al., 2011; O'Sullivan et al., 2011). Interestingly, miRNA-21, miRNA-100, miRNA-143 and miRNA-145 were also able to significantly discriminate between diffuse vs. focal ISR, yet, this last finding should be interpreted with attention as it originated from additional analyses performed on a fraction of the total sample, probably introducing bias such as loss of randomization i.e. cases and controls are no longer balanced groups or lesser power related to the smaller sample. Another recent report showed that miRNA-93-5p was differentially expressed between ISR and non-ISR patients, proposing miRNA-93-5p as a robust independent ISR predictor (O'Sullivan et al., 2019). Additionally, they found that the predictive performance of a model including main risk factors for ISR e.g., diabetes, stent length and diameter, together with common risk factors for CAD development such as age, sex, active smoking, diabetes, hypertension, and hyperlipidemia was further improved by adding miRNA-93-5p levels. Even though the results shown are encouraging, an important weakness of the study lies in the lack of additional validation in an independent cohort, restricting the extent of the results. Very recently, Dai et al. selected 14 angiogenesis-related candidate miRNAs (Dai et al., 2019) and reported 4 as independently associated with decreased restenosis risk (miRNA‐19a, miRNA‐126, miRNA‐210, and miRNA‐378). ROC curves showed that this subgroup of miRNAs had better predictive values for restenosis occurrence in Chinese population than each on its own (AUC: 0.776; 95% CI: 0.722‐0.831). Moreover, they found that 2 additional miRNAs (let‐7f and miR‐296) correlated with a lower risk of rapid angiographic stenotic progression (RASP), and together with the previous miRNAs, the model exhibited greater performance for RASP prediction (AUC: 0.879; 95% CI: 0.841‐0.917). Another similar study also performed in Chinese population recently reported that miRNA-146a and miRNA-146b were overexpressed in restenosis vs non-restenosis patients (P = 0.006), both holding prognostic value for restenosis risk in subjects with coronary heart disease (CHD) (Zhang et al., 2019). Analogously to the previous work, Zhang and colleagues also found that these miRNAs were up-regulated in RASP patients, and were both individually able to predict RASP occurrence in CHD subjects.

**Table 1:** Studies reporting extracellular miRNAs as restenosis biomarkers.

miRNA	Performance	95% CI	P	Sens. (%)	Spec. (%)	Expression in restenosis patients	Groups	Grouping	Stent type	Follow-up (years)	ISR	Sample	Ethnicity	Ref
miRNA-21	AUC: 0.568	0.372-0.757	<0.05	50.1	68.6	UR	51 ISR; 130 NISR; 52 Controls	Randomization	DES	0.5 - 1	Coronary	Plasma	Chinese	He et al., 2014
miRNA-100	AUC: 0.608	0.372-0.757	<0.05	60.2	68.9	DR
miRNA-143	AUC: 0.818	0.755-0.963	<0.001	82.1	80.1	DR
miRNA-145	AUC: 0.880	0.791-0.987	<0.001	88.7	83.1	DR
miRNA-93-5p	C: 0.734	not shown	0.0001	not shown	not shown	UR	39 ISR; 39 NISR	Consecutive, matched for age and diabetes	DES/BMS	4.9	Coronary	Plasma	Caucasian	O'Sullivan et al., 2019
miRNA-93 + FHSRF + SL and SD	C: 0.769	0.00001
miRNA‐19a, miRNA‐126, miRNA‐210 miRNA‐378	AUC: 0.776	0.722-0.831	not shown	not shown	not shown	DR	222 NISR; 64 ISR	Consecutive	DES	1	Coronary	Plasma	Chinese	Dai et al., 2019
miRNA-146a	AUC: 0674	0.567-0.781	not shown	not shown	not shown	UR	232 NISR; 23 ISR	Consecutive	DES	1	Coronary	Plasma	Chinese	Zhang et al., 2019
miRNA-146b	AUC: 0.801	0.729-0.875
miRNA-92a (TLR)	HR: 0.55	0.34-0.88	0.013	not shown	not shown	DR	26 primary endpoint; 21 secondary endpoint; 36 controls	Consecutive	not shown	1 and 2	PAD	Serum	Caucasian	Stojkovic et al., 2018
miRNA-195 (TLR)	HR: 0.40	0.23-0.68	0.001			
miRNA-195 (TVR)	HR: 0.40	0.22-0.75	0.005			
miRNA-92a + CF (TLR)	C: 0.70	0.60-0.80	0.130			
miRNA-195 + CF (TLR)	C: 0.75	0.66-0.85	0.030			
miRNA-143	AUC: 0.866	not shown	not shown	83.7	82.6	DR	74 ISR; 91 NISR	Consecutive	BMS???	not shown	LEAOD	Serum? Plasma?	Chinese	Yu et al., 2017
miRNA-21	AUC: 0.938	0.898-0.977	not shown	83.5	98.2	DR	79 ISR; 327 NISR	Consecutive, randomly chosen	BMS	0.5	LEAOD	Plasma	Chinese	Zhang et al., 2017
miRNA-320a	AUC: 0.766	not shown	not shown	82.1	63.8	UR	78 ISR; 68 NISR; 62 controls	Consecutive	Not shown	not shown	LEAOD	Plasma	Chinese	Yuan et al., 2019
miRNA-572	AUC: 0.690	69.2	68.9											

AUC, area under the curve; BMS, bare metal stent; C, C-statistic (a comparable measure to AUC); CF, clinical factors; DR, down-regulated; DES, drug eluting stent; FHSRF, Framingham heart study risk factors; HR, hazard ratio; ISR, in-stent restenosis; LEAOD, lower extremity arterial occlusive disease; NISR, non in-stent restenosis; PAD, peripheral artery disease; Sens., sensibility; Spec., specificity; SL, stent length; SD, stent diameter; TLR, target lesion restenosis; TVR, target vessel revascularization; UR, up-regulated.

In the case of peripheral artery disease (PAD) ISR, the role of 11 restenosis-related circulating miRNAs (miRNA-17, miRNA-21, miRNA-92a, miRNA-126, miRNA-143, miRNA-145, miRNA-195, miRNA-221, miRNA-222, miRNA-223, and miRNA-424) was examined in a primary endpoint constituted by target lesion restenosis (TLR) and atherothrombotic events, and a secondary endpoint represented by target vessel revascularization (TVR) (Stojkovic et al., 2018). Findings showed that miRNA-92a and miRNA-195 were independent predictors of the primary endpoint, but only miRNA-195 was able to independently predict TVR. Interestingly, miRNA-143 and miRNA-145 were detected at very low expression levels and were excluded from additional analyses even though they were previously suggested as ISR markers (He et al., 2014). Nonetheless, and similarly to the report from O'Sullivan and colleagues, adding miRNA-195 to clinical factors not only improved the ability to distinguish TLR from non-TLR subjects against a model considering miRNA-92a (P = 0.012), but also proved superior to a model integrating clinical risk factors plus both miRNA-92a and miRNA-195 (Stojkovic et al., 2018) (**Table 1**).

A series of studies exploring the utility of predictive miRNAs for lower extremity arterial occlusive disease (LEAOD) restenosis have been performed. One of them identified low levels of circulating miRNA-143 in restenosis vs. non-restenosis patients, correlating this measure with smoking status, history of diabetes, glucose, and low-density lipoprotein cholesterol (LDL-C) (Yu et al., 2017). Even though a low expression of the restenosis-related miRNA-143 is consistent with the findings from He and colleagues, it is unknown if the expression pattern remains the same for the rest of the previously reported restenosis-associated miRNAs, as Yu et al. evaluated miRNA-143 only. Additionally, it is also uncertain if the analysis was performed either in serum or plasma due to authors referred to both biological fluids as interchangeable concepts, an unfortunate but substantial ambiguity hampering a clear interpretation of the results and that will be later described. Another study showed overexpression of the coronary ISR-associated miRNA-21 in LEAOD restenosis patients (Zhang et al., 2017), constituting an excellent predictor of vascular restenosis according to ROC curve analysis, with an AUC of 0.938. Moreover, miRNA-21 was correlated with age, diabetes, and hypertension, and together with diabetes, miRNA-21 represented the main risk factors for LEAOD restenosis occurrence. Lastly, a very recent report found that circulating levels of miRNA-320a and miRNA-572 were significantly overexpressed in restenosis-developing LEAOD patients (Yuan et al., 2019). ROC curves also showed that these miRNAs were capable of discerning between patients developing ISR versus patients that not, with AUC values of 0.766 and 0.690, respectively. Although the results provided are auspicious, the sample size enrolled was relatively small. Additionally, the study fails to report minimal but very relevant clinical data like the stent types used, follow-up time, and important statistical estimates (**Table 1**), however, one of the strengths lies in the inclusion of a second control group made up by healthy volunteers besides the classical non-ISR group, a similar methodological approach than the study of He et al., allowing to better discriminate miRNA behavior between these 2 conditions. In this sense, it is noteworthy that the relative expression among miRNAs evaluated by Yuan et al. was very similar between the non-ISR group and healthy volunteers. Furthermore, 2 miRNAs showed comparable levels between ISR, non-ISR and healthy volunteers, which is also consistent with previous studies, and represents an interesting outcome if we consider that current extracellular miRNA normalization is commonly based on the addition of exogenous spike-in miRNAs, a technical issue that could be more suitably replaced by analyzing endogenous miRNAs stable enough for the discovery of restenosis biomarkers. Still, additional experimentation is needed to clarify this observation.

### Technical Challenges

Besides requiring a feasible and reliable analyte associated with a particular condition, miRNA routine sample analysis needs to cautiously overcome a significant amount of potentially detrimental obstacles that, if not properly managed, will not only affect miRNA analyses but most importantly, can compromise the patient's diagnosis and clinical management by inaccurate lab determinations. Consequently, before implementing miRNA measurement into day-to-day laboratory testing, a large number of technical issues must be correctly addressed. One of the very first concerns related to miRNA analysis comes from the collecting tubes employed for blood withdrawal. For instance, EDTA-collecting tubes can alter circulating miRNA detection, especially if samples are not immediately processed. Moreover, the longer it takes for sample processing, the stronger the effect on extracellular miRNA patterns (Leidinger et al., 2015). Studies show that proper attention must be paid when selecting the type of biological matrix for further miRNA analysis. For instance, serum samples were reported to contain a higher number of miRNAs than their corresponding plasma counterparts, even if analyzing the same individual, an outcome highly dependent on the measurement platforms used (Wang et al., 2012). However, various reports argue in favor of the opposing scenario, where not only plasma was reported to contain higher miRNA concentrations (McDonald et al., 2011), but miRNAs diversity was far more restricted in serum samples (Foye et al., 2017). Findings have also shown that serum- and plasma-abundant miRNAs such as miRNA-451a, miRNA-16-5p, miRNA-223-3p, and miRNA-25-3p are differentially expressed between these 2 biological fluids (Foye et al., 2017), reinforcing the idea that both fluids cannot be assumed to be interchangeable concepts regarding miRNA concentrations. Also, hemolysis affects directly the concentration of a small number of miRNAs (Kirschner et al., 2011; McDonald et al., 2011) which is consistent with reports showing particular and specific erythrocytes-derived miRNAs (Chen et al., 2008a; Kannan and Atreya, 2010). Deepening in this area, hemolysis was demonstrated to affect a far greater number of miRNAs than previously reported, compromising miRNAs previously recognized as important biomarkers for various diseases (Kirschner et al., 2013). Interestingly, the use of a ratio between the hemolysis-dependent and -independent miRNA-451 and miRNA-23a, respectively, has been proposed to better assess the degree of erythrocyte lysis and therefore, diminish the effect of hemolysis on blood-based miRNA determinations (Blondal et al., 2013).

On the other hand, important variations for biomarker discovery can be introduced at the analytical stage, which is highly dependent on the measurement platform selected. In the case of miRNAs, the most common and widely used technique corresponds to qPCR largely due to its robustness, relative ease, elevated specificity and sensitivity, broad dynamic range and high resolution, among others. However, each step required for qPCR assays can introduce a different cause of variation that can mask the biological differences we are looking to determine, and to date, several unsolved questions can affect circulating miRNA analysis when using this system. For example, to date, there is no predetermined or consensus set of extracellular miRNAs that can be used for normalization (Roberts et al., 2014), which would be the ideal scenario to allow proper comparisons between a target miRNA against a normalizer miRNA to obtain reliable miRNA expression levels. In contrast, nowadays normalization is frequently achieved by using synthetic alternatives such as exogenous miRNAs that are spiked in during RNA isolation in an attempt to avoid technical differences regarding the extraction procedure. Another different normalization strategy is the use of ncRNAs such as small nuclear RNAs (snRNA) or small nucleolar RNAs (snoRNAs), however, to select the proper normalizer, a set of these ncRNAs must be previously analyzed in each lab for validation purposes to obtain accurate results. Importantly, RNA quality is one the most fundamental determinants of reproducibility for qPCR results, and improper sample handling regarding the collection, transport or storage can affect RNA integrity and unambiguously lead to irreproducible experiments. Therefore, every RNA preparation must be meticulously assessed to ensure that nucleic acids present have not been degraded. In general, a standardized qPCR protocol should be closely followed to ensure consistency between diverse laboratories, as suggested in the MIQE guidelines (minimum information for publication of quantitative real-time PCR experiments) (Bustin et al., 2009).

### Future Perspectives

miRNA research as ISR biomarkers is still at an early stage. The scarce findings reported so far include conventional flaws in study designs such as small samples, lack of proper control groups or validation cohorts, and the absence of clinical, technical and statistical data that may be crucial for a correct interpretation and reproducibility. The tolerant operative consensus at the time of reporting putative restenosis biomarkers leads to inconsistent or unreliable candidate miRNAs, and to advance the field, investigations must meet minimal and uniform conditions. Clinical outcomes should be very well defined to turn them into quantifiable events, or at least easily measurable. In this sense, large randomized, multicenter, prospective trials capable of establishing whether miRNAs can effectively predict clinical features are greatly needed.

A persistent but reasonable shortcoming regarding miRNA research as biomarkers for restenosis is represented by the candidate approach, i.e., handpicking specific RNA molecules exclusively based on previous reports. Although most of these investigations have solid grounds since they are based on the choice of miRNAs previously associated with restenosis-related mechanisms, the success rates contrast with what one might expect, since they are not even close to 100%, as the case of different studies mentioned above (Stojkovic et al., 2018; Dai et al., 2019). The candidate approach is predominantly used because is significantly less expensive than other wide-ranging strategies, such as microarray or NGS, but the loss of information can be excessive. On the contrary, using, for example, NGS allows having the clearest depiction of the total amount of miRNAs that may be relevant or even participate in the endpoint and that we could be missing when executing the candidate methodology, which ultimately points to the cost-benefit relation.

Even though important technical difficulties can further delay the arrival of miRNAs into the clinic, ongoing research in the matter has allowed the most common problems to be properly identified and therefore, prone to correction with an adequate and strictly controlled standardization of laboratory practices, including pre-analytical, analytical and post-analytical procedures, eliminating as many possible variables affecting routine miRNA determinations. But even if we carefully consider the aforementioned arguments, the complete potential of miRNAs as clinically applicable biomarkers is rather far from becoming an imminent reality, as some additional and significant questions remain poorly explored, for example, the existence of circadian oscillations of human miRNAs. A recent and very stimulating line of research has demonstrated important diurnal variations in miRNA levels (Rekker et al., 2015; Heegaard et al., 2016; Hicks et al., 2018), with important and detrimental implications for an exceedingly trivial preanalytical issue such as establishing the proper moment to collect blood samples.

## Author Contributions

NV wrote sections of the manuscript. TZ contributed conception, design and wrote sections of the manuscript. FL and LS contributed critical analysis and wrote sections of the manuscript. All authors contributed to manuscript revision, read and approved the submitted version.

## Funding

This work was supported by FONDECYT-Chile (grant number 3170785).

## Conflict of Interest

The authors declare that the research was conducted in the absence of any commercial or financial relationships that could be construed as a potential conflict of interest.

## Abbreviations

AUC, area under the curve; AGO2, argonaute protein family 2; BMS, bare metal stent; CABG, coronary artery bypass grafting; CHD, coronary heart disease; CVD, cardiovascular disease; DES, drug-eluting stent; DGCR8, DiGeorge syndrome critical region 8; EC, endothelial cells; ECM, extracellular matrix; EDTA, ethylene diamine tetra-acetic acid; EGF, epidermal growth factor; FGF-2, fibroblast growth factor 2; IGF, insulin-like growth factor; ISR, in-stent restenosis; LEAOD, lower extremity arterial occlusive disease; LDL-C, low-density lipoprotein cholesterol; miRNA, micro ribonucleic acid; MAPK, mitogen-activated protein kinase; MIQE,minimum information for publication of quantitative real-time PCR experiments; mRNA, messenger ribonucleic acid; ncRNAs, non-coding RNAs; NGS, next-generation sequencing; PAD, peripheral artery disease; PCI, percutaneous coronary intervention; pre-miRNA, precursor micro ribonucleic acid; pri-miRNA, primary micro ribonucleic acid; PTCA, percutaneous transluminal coronary angioplasty;qPCR, quantitative polymerase chain reaction; RASP, rapid angiographic stenotic progression; RISC, RNA-induced silencing complex; ROC, receiver operating characteristic; Rnases, ribonucleases; snRNA, small nuclear RNAs; snoRNAs, small nucleolar RNAs; TLR, target lesion restenosis; TVR, target vessel revascularization; VSMC, vascular smooth muscle cells; XPO5, exportin 5; 3′-UTR, three prime untranslated region; 5′-UTR, five prime untranslated region
